# Chicago Medical Society

**Published:** 1885-04

**Authors:** Liston H. Montgomery

**Affiliations:** Secretary


*Meeting of March 2, 1885.*—The President, Dr. D. A. K. Steele, in the chair.

*Mollities Ossium.*—Dr. W. J. Webb read a paper on this disease, embodying an elaborate account of the case of a man, a native of Pennsylvania, some of whose brothers and sisters had had symptoms more or less like his own, so that an hereditary element might have entered into the causation of the disease. He had led a life of vicissitudes, having been at different times a wood-chopper and a miner; had been a masturbator from the age of eight years; had at times been a hard drinker; was supposed to have had syphilis; and perhaps had been poisoned by drinking arsenical water while engaged in mining. He had also suffered from tape-worm. Toward the close of his life his body became very much distorted; he was at one time unable to move any part of his person except his right hand, he was cyanosed, and on several occasions his temperature was subnormal.

At the post-mortem examination, the body was found so shortened and flattened as to resemble that of a frog; yet it was six or seven inches longer than it had been before death. It was excessively emaciated, and the skeleton was distorted in various ways. After being subjected to a stretching process, the entire length of the cadaver was fifty-five inches. The left femur measured eleven inches, the right one eleven and three quarters; the left tibia fourteen inches, and the right one thirteen and a quarter; the sternum six and a half; each humerus eleven; and the left ulna nine, and the right one ten. The heart was situated in the median line, and the pericardium extended up to the second rib. The head was at least an inch and a quarter smaller in circumference than it had formerly been. The vault of the cranium was nearly as flexible as a moderately distended foot-ball. The bones of the face had suffered least of all, but they had been tender to the touch before death, and some of the teeth had become loose. The clavicles were enlarged, elongated, and curved decidedly downward. The sternum was but little affected; the manubrium, together with the sternal ends of the clavicles, extended upward and forward, while the gladiolus pointed upward and slightly backward, which made a decided curve posteriorly. In the latter third of the shafts of the ribs nearly or quite all the proper bone substance was wanting, and the periosteum was folded upon itself. Altogether, the condition of the chest-wall was such as to have caused great compression of the organs within, and for at least six months before the man's death his breathing had been largely diaphragmatic.

The vertebral column was enlarged, and, while its natural curves were increased, there was also a decidedly sharp lateral curvature to the right in the dorsal portion, together with a slight curve to the left in the lumbar portion. The innominate bones were nearly at right angles with the horizontal plane of the pelvis. The ilia were crowded inward, while the ischia were directed outward and the sacrum was turned forward, and the pubes slightly backward and downward. This position of the pelvis, together with the eversion and outward curvature of the femora, was suggestive of the ancient lyre. The upper limbs presented nothing of special importance, except that they were small and so soft and flexible that they might easily have been tied in a knot. On section of the femur, only a trace of bone substance was seen adhering to the inner surface of the periosteum; as the head of the bone was approached, the cancellated structure was more characteristic, but even here it could easily be cut with an ordinary scalpel. When the periosteum was cut into, a considerable quantity of sero-sanguineous fluid escaped that completely enveloped and seemed to permeate the liver-like substance which had taken the place of the medullary canal and the greater part of the shaft of the bone. In the trochanters there was an augmentation of soft cancellated substance containing a large deposit of fat. The neck of the bone was shortened, and the great trochanter extended above the articulation with the acetabulum.

The reader then gave a summary of the treatment to which the man had been subjected, and suggested that, when practicable, a water-bed should be used for such patients, while something might perhaps be done with an apparatus for making extension and preserving the proper shape of the thorax—possibly with plaster of paris. According to an old author (who was quoted at length), cases of osteomalacia might be divided into three classes: 1. Those due to carcinomatous or sarcomatous disease of the medulla. 2. Those due to an affection of the medulla, allied to lymphoma. 3. Those due to some peculiar condition of the medulla, or perhaps of the blood, dependent on the pregnant state. The reader thought that hereafter the nervous system would receive more attention than it had received hitherto in connection with this disease.

Dr. C. E. Webster asked if the reader, in his study of the disease, had been able to distinguish it sharply from fragilitas ossium. It seemed to him that the two were often confounded. He doubted if any form of support could have been devised that would prevent the deformity of the thorax.

*Typhoid Fever and its Hygienic and Specific Treatment* was the subject of a paper by Dr. David O'Shea, which was illustrated by the histories of a number of cases. Foul air and contaminated drinking-water were not always the cause of the disease, but he believed that there was a specific enteric bacillus yet to be discovered. Klebs had already professed to have identified the bacillus, and to have found it in the blood, in the lymphatics, and in the tissues, but this needed confirmation. It was very rare for the disease to occur in a person far advanced in years, but the individuals of certain families were prone to suffer from it in a severe form, so that there was reason to apprehend a formidable attack when a parent had died of it. When we were called to a suspected case, therefore, we should first consider the patient's family history, then his personal history, and finally his actual condition, with regard to their bearing on the prognosis.

The principles of treatment were generally accepted and well understood, and success would depend greatly on their intelligent application to individual cases and on careful attention to details in every stage of the disease. Whenever it was possible, he had the patient's bed placed so that, occupying a large, airy room, with the doors or windows open more or less continuously, according to the state of the weather, it could be approached from either side. The bed should be firm and comfortable, and, if practicable, one should be occupied during the day and another at night. The covering should be light, but sufficient to protect the patient against changes of temperature. The body should be sponged night and morning with tepid alcohol and water, and during the height of the fever the face and hands should be sponged frequently during the day. If the temperature rose above 103° F. in the morning, and above 104° in the evening, the wet pack should be applied to the chest and an ice-bag to the head, and a mustard foot-bath three times in the twenty-four hours would prove very grateful and efficacious. The bed-pan should always contain about one-third its capacity of water with a teaspoonful of a twenty-five per-cent. solution of carbolic acid added to it, and this should be renewed every time the vessel was used. In summer the evacuations should be buried, but in winter they could be thrown into a water-closet. No one should occupy the same bed with the patient, nor, if it could be avoided, the same room, and only those who were advanced in years should act as nurses. In all the cases related, this hygienic treatment had been followed, together with the specific treatment laid down by Liebermeister, Traube, Wunderlich, and others, consisting in the administration of ten grains of calomel at a single dose the first day the fever was recognized, and eight grains a day for three or four days afterward, or according to the condition of the bowels. It was a fact that these large doses of calomel had an antipyretic effect in every case, and, with this remedy, the writer could bear testimony to the efficiency of the specific treatment in cutting the disease short and lessening its mortality. The longest duration of the disease in any of his cases was twenty-six days, and the shortest fifteen days. The complications were few and of short duration; only one of the patients became delirious, and in that case the disease had not been recognized until the beginning of the second week, so that several days had elapsed before any calomel was given. Whether or not this fact was the cause of the delirium, he would not undertake to say—he merely mentioned it. The same patient was sick the longest time, and had the most copious evacuations. The true value of this specific plan of treatment could not be arrived at from such a limited number of cases, but he could recommend its further trial.

Dr. G. C. Paoli thought we could rely on nature in light cases, and that such cases served to explain the temporary popularity of the various specific plans of treatment that had sprung up at one time and another and been abandoned. He had seen cases run their course in eight, nine, or ten days, and he had seen others continue for twenty, twenty-five, thirty, or forty days; again, he had seen the disease in a malignant form go on to a fatal termination speedily. He relied a good deal on nourishment and careful nursing. He thought there was danger in the plan of using two beds, as suggested by the author, for, when Peyer's glands were inflamed, or when their cicatrization was incomplete, moving the patient from one bed to another might lead to trouble. The diarrhœa of typhoid fever could not be stopped, but we could control it within certain limits. He did not believe that there was such a thing as a typhoid-fever bacillus, or that one would ever be discovered. He gave but little quinine in the disease, and had seen two patients die from the administration of forty grains of the drug during an attack.

Dr. C. G. Davis was glad to see prominence given to the hygienic plan of treatment; formerly, under the notion of scourging the disease out of the system, too much medicine had doubtless been given. His observation had been that the mortality varied in different years and in different localities; he had seen it as high as twenty per cent. and as low as four per cent. A favorite piece of medication with him was to give a twenty-fourth of a drop of tincture of nux vomica and a sixth of a drop of tincture of aconite-root every four hours; this lessened the tendency to irritation of the gastro-intestinal mucous membrane, and he did not regard it as homœopathic.

Dr. C E. Webster usually followed the expectant plan. He sometimes ordered brandy in teaspoonful doses, as occasion required.

Dr. R. Randolph gave a minute description of his method of applying cold water. An oil-cloth was placed under the patient, after his night-shirt had been pulled up over his head, and then cool water was pressed out of a sponge over the surface of the body. The oil-cloth was easily removed when the process was over. He had never seen quinine reduce the temperature; on the contrary, he believed that it acted as a cardiac depressant.

Dr. G. W. Webster could speak of cold baths from personal experience, having had typhoid fever himself. The baths that he was subjected to produced very severe shock, so that, after having had two or three of them, he had refused to submit to any more. Moreover, they did not reduce his temperature more than a tenth of a degree. Sponging with tepid water, however, was such a pleasure that he looked forward to it eagerly. He was given large doses of quinine, which so weakened his heart that he did not recover from the effects for as much as a year. He had seen salicylate of bismuth used very successfully in a number of cases.

Dr. Randolph remarked that more attention was now given to the temperature than to the pulse, and that the observation of the pulse was getting to be one of the lost arts.

Dr. G. W. Webster called attention to the secondary dilating action of cold upon the superficial capillaries, and instanced the burning sensation felt in the hands soon after handling snow or ice.

Dr. Angear doubted if this observation would apply to the moderate cold that was used in the treatment of fevers.

Dr. G. Newkirk did not think it necessary to apply cold to reduce the temperature. The warm pack imparted a pleasant sensation of quiet and rest, and kept the skin active, which was an essential point in the treatment.

The paper was further discussed by Dr. R. E. Starkweather, the President, and Dr. O'Shea.

LISTON H. MONTGOMERY, M. D., *Secretary.*

